# Superior GVHD-free, relapse-free survival for G-BM to G-PBSC grafts is associated with higher MDSCs content in allografting for patients with acute leukemia

**DOI:** 10.1186/s13045-017-0503-2

**Published:** 2017-07-04

**Authors:** Qian Fan, Hui Liu, Xinquan Liang, Ting Yang, Zhiping Fan, Fen Huang, Yiwen Ling, Xin Liao, Li Xuan, Na Xu, Xiaojun Xu, Jieyu Ye, Qifa Liu

**Affiliations:** 1 Department of Hematology, Nanfang Hospital, Southern Medical University, No. 1838 North Guangzhou Avenue, Guangzhou, 510515 China; 2 Department of Hematology, First People’s Hospital of Chenzhou, No. 102 Luojiajing District, Chenzhou, 423000 China; 3 Department of Hematology, Concord Hospital of the Fujian Medical University, No. 29 Xinquan Road of Gulou District, Fuzhou, 350001 China; 4 Department of Hematology, People’s Hospital of Zhongshan City, No. 2 Zhongshan Road, Zhongshan, 528403 China

**Keywords:** Granulocyte colony-stimulating factor (G-CSF)-primed bone marrow, Graft-versus-host disease, GVHD-free/relapse-free survival, Myeloid-derived suppressor cells

## Abstract

**Background:**

Granulocyte colony-stimulating factor (G-CSF)-mobilized peripheral blood stem cells (G-PBSC) has largely replaced unstimulated bone marrow (un-BM) for allografting because of accelerated engraftment, but with a higher morbidity and mortality of graft-versus-host-disease (GVHD). Recent studies suggested that G-CSF-primed BM (G-BM) had similar engraftment but lower morbidity and mortality of GVHD comparing to G-PBSC. A prospective, randomized, multicenter study was conducted to compare G-BM with G-PBSC as the grafts in allogeneic hematopoietic stem cell transplantation (allo-HSCT) for acute leukemia in first complete remission (CR1).

**Methods:**

Totally 101 adult leukemia in CR1 undergoing HLA-identical sibling transplants were randomized into G-BM or G-PBSC group. The primary study endpoint was GVHD-free/relapse-free survival (GRFS).

**Results:**

Both the engraftment of neutrophil and platelet were 2 days later in G-BM than in G-PBSC group (*P* = 0.412, *P* = 0.39). G-BM group showed significantly lower II–IV acute GVHD (aGVHD) and similar III–IV aGVHD compared with G-PBSC group (12.2% vs 28.8% for II–IV, *P* = 0.048; 4.1% vs 9.6% for III–IV aGVHD, *P* = 0.267, respectively). The overall cumulative incidence of chronic GVHD (cGVHD) at 3 years were 22.3% ± 6.3% and 44.8% ± 7.6% (*P* = 0.026), respectively, and extensive cGHVD were 4.5% ± 3.1% and 15% ± 5.3% (*P* = 0.08), respectively, in G-BM and G-PBSC groups. Two groups had similar 3-year relapse, transplant-related mortality (TRM), overall survival (OS), and disease-free survival (DFS) (all *P* > 0.05). G-BM group showed significantly higher probability of GRFS than G-PBSC group (73.5% ± 6.3% vs 55.8% ± 6.9% at 1 year, *P* = 0.049; 69.0% ± 6.7% vs 49.7% ± 7.0% at 2 and 3 years, *P* = 0.03, respectively). Graft content analysis revealed statistically higher frequency of myeloid-derived suppressor cells (MDSCs) in the G-BM than in G-PBSC grafts (*P* < 0.01), and recipients received statistically higher numbers of MDSCs in G-BM than in G-PBSC group (*P* = 0.045). Numbers of MDSCs infused to patients were negatively correlated with the severity of aGVHD (*P* = 0.032, *r* = −0.214). Multivariate analysis showed that MDSC cell dose below the median (HR = 3.49, *P* < 0.001), recipient age (HR = 2.02, *P* = 0.039), and high risk of disease (HR = 2.14, *P* = 0.018) were independent risk factors for GRFS.

**Conclusions:**

G-BM grafts lead a better GRFS and less GVHD associated with a higher MDSCs content compared with G-PBSC grafts.

## Background

Granulocyte colony-stimulating factor (G-CSF)-mobilized peripheral blood stem cells (G-PBSC) has replaced bone marrow (BM) as the most commonly used source of hematopoietic stem cells (HSCs) because of faster engraftment and practicability as well as acceptable graft-versus-host disease (GVHD) [1]. Even though, some studies suggested that G-PBSC transplants might result in a higher morbidity and mortality of GVHD, especially chronic GVHD (cGVHD), compared with unstimulated BM transplants, as G-PBSC grafts contain 4- to 10-fold more T lymphocytes [2, 3]. Recently, several prospective and retrospective studies suggested that G-CSF-primed BM (G-BM) grafts had similar engraftment but lower morbidity and mortality of GVHD compared with G-PBSC grafts [4].

The mechanisms of G-CSF-primed grafts inducing immune tolerance are extensively studied, which include the induction of T helper type 2 (Th2) cell polarization and increase of CD4^+^CD25^+^ regulatory T (Treg) cells and tolerogenic dendritic cell differentiation [5–8]. But, these mainly focused on PBSC grafts. The mechanisms of G-BM grafts inducing immune tolerance are not fully understood and whether G-CSF has similar impact on BM or other mechanisms exist in G-BM associated immune tolerance was rarely studied.

Each graft source has its unique compositions, such as the number of CD34^+^ cells, different T cell subtypes and natural killer (NK) cells and, possibly, other cellular as well as cytokine components, which affect the time frame of engraftment and incidence of GVHD [9, 10]. Myeloid-derived suppressor cells (MDSCs) are a heterogeneous population of myeloid progenitors and immature myeloid cells (IMC) with a potent immunosuppressive activity, which establish an important role in the tumor immune responses [11, 12]. Recently, a few studies began to focus on the role of MDSCs in the graft immune tolerance. In animal model, Highfill et al. reported that co-transplantation of MDSCs might decrease the severity and mortality of aGVHD [13]. In human, Vendramin et al. documented that the numbers of MDSCs in the grafts were negatively correlated with incidence of aGVHD [14]. Our previous study showed that G-CSF might induce the expansion of MDSCs in the BM and peripheral blood (PB) in vivo and higher frequency of MDSCs consisted in G-BM grafts than G-PBSC grafts [15].

Based on these study results, we hypothesized that G-BM grafts lead a better GVHD-free/relapse-free survival (GRFS) and less GVHD associated with a higher MDSC content compared with G-PBSC grafts except for T cells and other compositions. And MDSCs might play the immunoregulatory role in GVHD. To identify this hypothesis, a randomized multicenter clinical trial was conducted.

## Methods

### Study design and patients accrual

This prospective, randomized, open-label study was conducted from February 2013 to April 2015 in Nanfang Hospital, Southern Medical University, the First People’s Hospital of Chenzhou, People’s Hospital of Zhongshan City, and Concord Hospital of the Fujian Medical University. One hundred one patients with acute leukemia in first complete remission (CR1) undergoing allogeneic stem cell transplantation from an HLA-identical sibling were enrolled in this trial of G-BM or G-PBSC as the source of stem cells. The study was performed in accordance with modified Helsinki Declaration, and the protocol was approved by respective ethical review boards before study initiation. All patients and donors provided written informed consent.

### Conditioning regimen and GVHD prophylaxis

As we described previously [16], two myeloablative conditioning regimens were used, including BuCy (busulfan + cyclophosphamide) and TBI (total body irradiation) + Cy. Generally, acute myeloid leukemia (AML) received BuCy and acute lymphoid leukemia (ALL) TBI + Cy. Cyclosporine and short-course methotrexate were given as GVHD prophylaxis [16].

### Supportive therapy and infection prophylaxis

As described previously [16], low molecular weight heparin and prostaglandin E were used from the beginning of the conditioning to engraftment for hepatic veno-occlusive disease (HVOD) prophylaxis. Phenytoin orally was used for the prophylaxis of busulfan toxicities on the central nervous system. Oral sulfamethoxazole were used for prophylaxis of *Pneumocystis carinii*. Acyclovir and ganciclovir were given for prophylaxis and treatment of cytomegalovirus (CMV) infection as prescribed in previous literature [16]. Antifungal agents were administered according to the history of invasive fungal infection (IFI) or not. Generally, all patients received granulocyte colony-stimulating factor (G-CSF, 5 μg/kg/day) from +3 days post-transplantation until achievement of the peripheral white blood cells count reached 1.0 × 10^9^/L or absolute neutrophil count (ANC) reached 0.5 × 10^6^/L. Patients received red blood cells and platelet transfusions if hemoglobin levels were ≤70 g/L and platelet count ≤20.0 × 10^9^/L.

### Stem cell mobilization and collections

All donors received G-CSF (filgrastim, Kirin Brewery Co, Tokyo, Japan) 5 μg/kg/day for 5 days. G-BM harvest was performed on the fifth day (volume, 15–20 mL/kg patient adjusted ideal weight). At least 3.0 × 10^8^ total nucleated cells/kg or 2 × 10^6^ CD34^+^ cells/kg recipient ideal body weight was collected. G-PBSC harvest was performed from day 5 of G-CSF to obtain 6.0 × 10^8^ total nucleated cells/kg or a minimum CD34^+^ cell count of 3 × 10^6^/kg recipient ideal body weight.

### Flow cytometry analysis in grafts

Two grafts were analyzed in terms of CD34^+^ cells, CD3^+^ T cells and subpopulation (CD3^+^CD4^+^, CD3^+^CD4^+^CD45RA^+^, CD3^+^CD4^+^CD45RO^+^, CD3^+^CD8^+^, CD3^+^CD8^+^CD45RA^+^, CD4^+^CD25^+^FOXP3^+^ Treg cells), CD19^+^B cells, CD3^−^CD56^+^ NK cells, and MDSCs by flow cytometry using standard procedures. MDSCs were defined as Lin^low/neg^HLA-DR^−^CD33^+^CD11b^+^. All results were assayed by BD FACSCanto TMII (BD Biosciences), and the acquired data were further analyzed using BD-FACSDiva Software. Flow cytometric results were represented as percentage positive.

### MDSCs suppression assay

Purified CD3^+^ lymphocytes and MDSCs were isolated from G-BM or G-PBSC grafts by CD3 and HLA-DR, CD33 microbeads (Milenyi Biotec, Bergisch Gladbach, Germany), according to manufacturer’s instructions. Purity of the selected populations was evaluated by flow cytometry, demonstrating an efficiency of separation above 95% in all experiments. Purified CD3^+^ T lymphocytes were labeled with 5 μM 5, 6-carboxy-fluorescein diacetate succinimidyl ester (CFSE) (Biolegend) and thereafter were co-cultured with isolated MDSCs at a MDSCs-to-CD3^+^ T cell ratio of 1:1 and 1:5 in the presence of anti-CD2/CD3/CD28 biotin beads (Miltenyi Biotec) in RPMI 1640 (Sigma-Aldrich) supplemented with 10% FBS, penicillin/streptomycin, and 2 mM l-glutamine. For positive control, lymphocytes were stimulated in the absence of MDSCs; for negative control, CD3^+^ cells and MDSCs were incubated without the stimuli. After 4 days of culture, the cells were harvested for CFSE dilution analysis by flow cytometry.

### Endpoints and definitions

The primary study endpoint was GRFS. Secondary study endpoints were hematopoietic engraftment, acute and chronic GVHD, early infections, disease relapse, transplant-related mortality (TRM), overall survival (OS), and disease-free survival (DFS). GRFS was defined as absence of grade III–IV aGVHD, systemic immunosuppressive therapy requiring cGVHD, relapse, or death for any causes after allogeneic hematopoietic stem cell transplantation (allo-HSCT) [17, 18]. Hematopoietic engraftment was defined as the first of two consecutive days with an absolute neutrophil count in the peripheral blood exceeding 0.5 × 10^9^/L and the first of 3 days with an absolute platelet count exceeding 20 × 10^9^/L without transfusion support. Complete chimerism was defined as >95% donor cells detected. aGVHD was graded according to standard criteria [19], and cGVHD was assessed in patients surviving for more than 100 days post-transplantation and defined as limited or extensive [20]. Relapse was defined by molecular, cytogenetic, or morphologic evidence of the original hematologic disease in the peripheral blood, BM, or any extramedullary site. TRM was defined as death from any cause other than relapse. DFS was defined as survival in a state of continuous complete remission.

### Statistical analysis

Analysis was performed on October 31, 2016. Comparisons of categorical variables were made by means of chi-squared and Fisher’s exact tests for small numbers. Differences between numerical variables were calculated by means of the Mann-Whitney *U* test. Numerical variables were analyzed as categories based on their values being below or above the median of the entire cohort. Correlation analysis between categorical variables was calculated using Spearman’s correlation test. Incidence of time-dependent outcomes was estimated by the method of Kaplan-Meier and compared by the log-rank test. Cumulative incidence curves in a competing risks setting were used to calculate probabilities of relapse and TRM. Cumulative incidence of relapse were calculated by regarding death as the competing event, whereas estimates of TRM were calculated by regarding disease relapse as the competing event [21]. Gray test was used to compare the incidence of TRM and relapse between groups [22, 23]. Cox proportional hazards model was used to evaluate the associations of patient and graft characteristics with various outcomes. Factors that were tested in the multivariable analyses were patient’s age, gender, the source of grafts, primary disease, risk classification, CD3^+^ T cell dose, Treg cell dose, and MDSC dose. All statistical tests were two-sided, and a *P* value less than 0.05 was used to indicate statistical significance.

## Results

### Patients and transplant characteristics

A total of 101 patients were enrolled, with 49 randomized to G-BM and 52 to G-PBSC group. The primary diseases included acute myelogenous leukemia (AML, *n* = 65) and acute lymphoblastic leukemia (ALL, *n* = 36). Patients were all in CR1 and received grafts from matched siblings. The characteristics of patients, donors, and transplants are summarized in Table 1. There were no significant differences in patients’ age, gender, primary disease, and conditioning regimen between the two groups (Table 1, all *P* > 0.05). G-PBSC recipients received statistically higher numbers of total nucleated cells (TNC), CD34^+^ cells, and about 4-fold more CD3^+^ T cells per kilogram than G-BM recipients (Table 1, all *P* < 0.01).

**Table 1:** Patient, donor, and transplant characteristics

	G-BM (*n* = 49)	G-PBSC (*n* = 52)	*P* value
Patient age, median(range), year	30(13–59)	33(16–48)	NS
Male patients (%)	24(48.9)	28(53.8)	NS
Underlying diseases (%)			
AML	34(69.4)	34(65.4)	NS
ALL	15 (30.6)	18(34.6)	NS
Risk classification (%)			
Standard risk	26(53.1)	27(51.9)	NS
High risk	23(46.9)	25(48.1)	NS
Donor age, median(range), year	34(10–20)	38(17–55)	NS
Male donors (%)	25(51.0)	28(53.8)	NS
Female to male (%)	16(32.6)	18(34.6)	NS
Median TNC, 10^8^/kg (range)	5.8(3.8–8.2)	8.7(7.2–12.5)	*P* < 0.01
Median CD34^+^ cell count, 10^6^/kg (range)	3.6(2.3–10.0)	5.6(3.5–12.3)	*P* < 0.01
Median CD3^+^ cell count, 10^6^/kg (range)	69.6(22–156)	285.8(68–467)	*P* < 0.01

*ALL* acute lymphoblastic leukemia, *AML* acute myelogenous leukemia, *TNC* total nucleated cells

### G-CSF induces an expansion of MDSCs in vivo

**Fig. 1:**
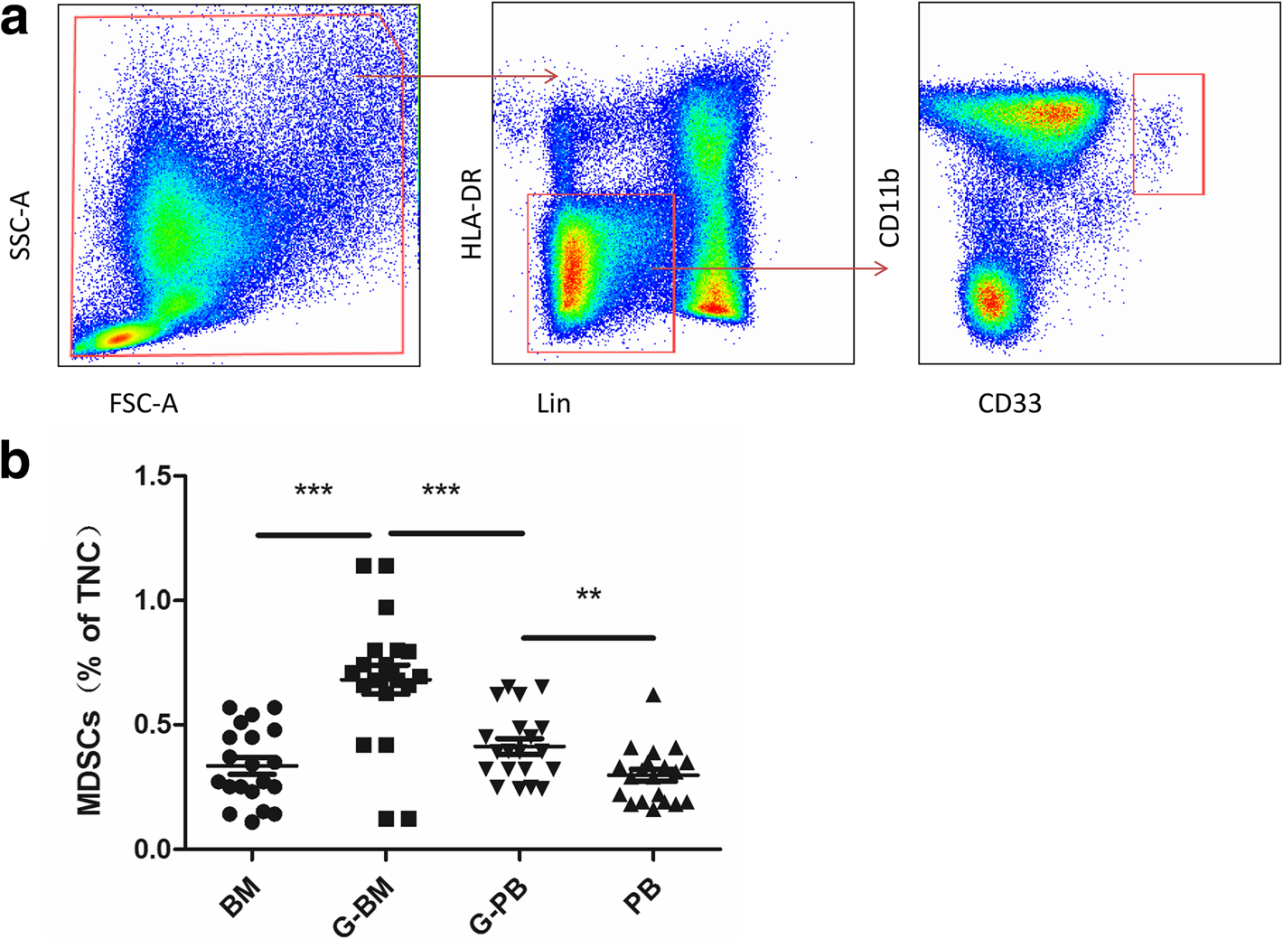
Expression of MDSCs in the BM and PB before and after G-CSF mobilization. **a** Sequential gating strategy for MDSC identification (Lin^low/neg^HLA-DR^−^CD11b^+^CD33^+^) using flow cytometry. **b** Cumulative dot plots showing MDSC frequencies from BM and PB of 20 donors before and after G-CSF mobilization (**P < 0.01, ***P < 0.001)

To identify whether G-CSF induces the expansion of MDSCs in vivo, 20 donors were studied for the frequencies of MDSCs in the BM and PB before and after G-CSF mobilization (day 5). MDSCs were quantified with the following gating strategy (Fig. 1a). The results showed, before G-CSF mobilization, the frequencies of MDSCs in the BM and PB were 0.33% ± 0.15% and 0.30% ± 0.13% of total nucleated cells, respectively (Fig. 1b, *P* = 0.32). After 5 days mobilization, the frequency of MDSCs in the BM increased to 0.65% ± 0.29%, compared with 0.41% ± 0.17% in the G-PB (Fig. 1b, *P* < 0.01). Both in the BM and PB, the frequencies of MDSCs significantly increased after G-CSF treatment (Fig. 1b, both *P* < 0.05).

### Immunosuppressive activity of MDSCs

**Fig. 2:**
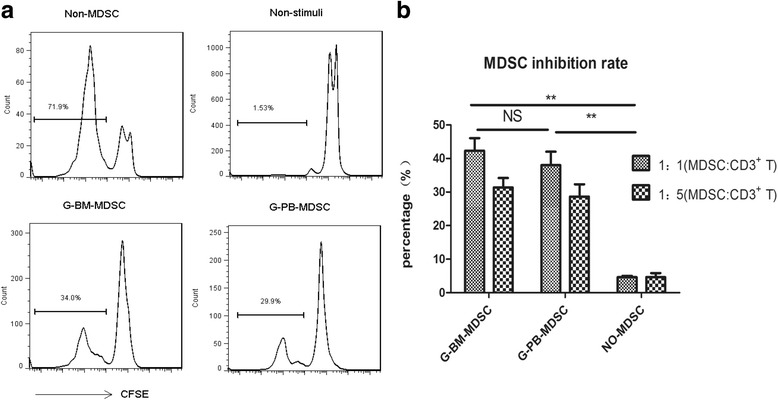
Suppressive activity of MDSCs isolated from G-BM and G-PBSC grafts. **﻿﻿a** The proliferation of purified and CFSE-labled CD3+ T cells in the coculture with isolated MDSCs or not. **b** The inhibition rate of the prolif﻿erati﻿on of CD3+ T cells by MDSCs. Isolated MDSCs were added at a 1:1 and 1:5 ratio to purified CD3+ T cells in the presence of anti-CD2/CD3/CD28 biotin beads according to manufacturer instructions (Miltenyi Biotec). Data are representative of three independent experiments (NS *P* > 0.05, **P < 0.01)

To verify whether G-CSF inducing MDSCs could be defined as functional MDSCs, we determined their immunosuppressive activity. Highly purified MDSCs from G-BM and G-PBSC grafts were, respectively, co-cultured with autologous CFSE-labeled CD3^+^ T cells for 4 days in the presence of T cell stimulators. The results showed that there was a significant inhibition of T cell proliferation in co-culture with MDSCs from the two grafts, which indicated that MDSCs from both G-BM and G-PBSC grafts were immunosuppressive (Fig. 2a, b). Further analysis revealed that the immunosuppressive activity of MDSCs was similar in the two grafts (Fig. 2b, *P* = 0.67).

### Graft content analysis

The percentage and absolute numbers of MDSCs, Treg cells and CD3^+^ T cells and subsets, CD19^+^ B cells, and CD3^−^CD56^+^ NK cells in the grafts for all the donors (49 in G-BM; 52 in G-PBSC group) were summarized in Table 2. As a result, the percentage of CD3^+^ T cells among lymphocytes was significantly lower in the G-BM than that in the G-PBSC grafts (*P* = 0.007). And the frequency of MDSCs was statistically higher in the G-BM than in the G-PBSC grafts (0.66% ± 0.23% vs 0.40% ± 0.19% of total nucleated cells, respectively, *P* < 0.01). The percentage of Treg cells in the G-BM was also higher than in the G-PBSC though without statistical significance (*P* = 0.192). The percentage of other cell subsets in the two groups did not differ significantly (all *P* > 0.05). The absolute numbers of MDSCs infused in the G-BM group was 1.38–7.31 × 10^6^/kg (median, 3.93) compared with 1.65–5.98 × 10^6^/kg (median, 3.53) in the G-PBSC group (*P* = 0.045). Cell doses of other compositions including CD3^+^ T cells were all significantly lower in the G-BM than in the G-PBSC group, which were partly associated with significantly less TNC infusion in G-BM than in G-PBSC group.

**Table 2:** G-BM and G-PBSC graft cell subset frequencies and cell doses

	Frequencies of graft cell subsets, (%), mean ± SD			Doses of graft cell subsets infused, 10^6^ cells/kg of body weight, median (min–max)		
	G-BM (*n* = 49)	G-PBSC (*n* = 52)	*P*	G-BM (*n* = 49)	G-PBSC (*n* = 52)	*P*
CD3^+^	60 ± 10	68.0 ± 14.0	0.007	69.6(22–156)	285.8(68–467)	<0.001
CD3^+^CD4^+^	29 ± 8.7	35.0 ± 10.7	0.065	33(10–56)	139.3(45–257)	<0.001
CD4^+^CD45RA^+^	9.5 ± 5.7	13.1 ± 8.1	0.220	11(3–30)	51.3(16–121)	<0.001
CD4^+^CD45RO^+^	13.8 ± 7.7	16.2 ± 8.0	0.647	16(5–39)	63.6(16–150)	<0.001
CD3^+^CD8^+^	26.6 ± 7.4	27.9 ± 9.2	0.297	30(25–59)	111.8(28–265)	<0.001
CD8^+^CD45RA^+^	19.6 ± 6.6	20.5 ± 7.6	0.625	22(11–60)	79.0(20–189)	<0.001
CD4^+^/CD8^+^	1.04 ± 0.5	1.12 ± 0.5	0.967			
CD19^+^	12.3 ± 5.0	12.7 ± 6.0	0.659	14(5–42)	48.5(12–90)	<0.001
CD3^−^CD56^+^	10.0 ± 7.0	7.0 ± 8.9	0.144	12(6–38)	27.9(3–76)	<0.010
Treg	3.1 ± 3.2	2.3 ± 2.0	0.192	3.0(1.5–6.7)	8.6(3.6–12.6)	<0.010
MDSCs	0.66 ± 0.23	0.40 ± 0.19	<0.01	3.9(1.4–7.3)	3.5(1.7–6.0)	0.045

CD3^+^ cell and subsets, CD19^+^ cell, and CD3^−^CD56^+^ cell frequencies are shown as percentage of the lymphocytes, CD4^+^CD25^+^FOXP3^+^ Treg frequencies are shown as percentage of CD3^+^CD4^+^ cells, and MDSC frequencies are shown as percentage of total nucleated cells

### Engraftment

Analyses of chimerism revealed that all patients obtained full donor chimerism by day +30 post-transplantation. Median time to neutrophil reconstruction was 14 days (range, 8–23 days) and 12 days (range, 8–19 days), respectively (*P* = 0.412), in the G-BM and G-PBSC groups. Median time to platelet reconstruction was 15 days (range, 9–87 days) and 13 days (range, 9–30 days), respectively (*P* = 0.390), in the G-BM and G-PBSC groups. To compare with unstimulated BM, the historical data of 32 patients who underwent unstimulated BM transplantation in our institutions between 1997 and 2003 was analyzed. The results showed that both the neutrophil and platelet reconstruction were significantly faster in the G-BM and G-PBSC than those in the unstimulated BM (G-BM, *P* = 0.023, *P* = 0.014, respectively; G-PBSC, *P* = 0.019, *P* = 0.009, respectively) with a median time of 22 days (range,16–36 days) to neutrophil reconstruction and 27 days (range, 18–46 days) to platelet reconstruction in unstimulated BM group.

### GVHD

Six patients developed II–IV aGVHD in the G-BM group, 15 patients in the G-PBSC group. The cumulative incidence of II–IV aGVHD was 12.2% ± 4.7% in the G-BM group compared to 28.8% ± 6.3% in the G-PBSC group (*P* = 0.048), and III–IV aGVHD was 4.1% ± 2.8% in the G-BM group compared to 9.6% ± 4.1% in the G-PBSC group (*P* = 0.267). Ninety-five patients were alive at day +100 post-transplantation, cGVHD occurred in 10 patients in the G-BM group, and 20 patients in the G-PBSC group, including 4 cases and 8 cases persistent from aGVHD, respectively. The overall cumulative incidences of cGVHD at 3 years were 22.3% ± 6.3% and 44.8% ± 7.6% (*P* = 0.026), respectively, and extensive cGHVD were 4.5% ± 3.1% and 15% ± 5.3% (*P* = 0.08), respectively, in G-BM and G-PBSC groups. Two patients died from cGVHD in G-PBSC group, while no patients died from cGVHD in G-BM group (*P* > 0.05).The comparison with historical data of 32 patients who underwent unstimulated BM transplantation in our institutions was also conducted. The results showed a trend of lower incidence of GVHD in G-BM comparing to that in unstimulated BM group (II–IV aGVHD, 12.2% vs 26.78%, *P* = 0.070; overall cGVHD 22.3% vs 37.5%, *P* = 0.120, respectively).

**Fig. 3:**
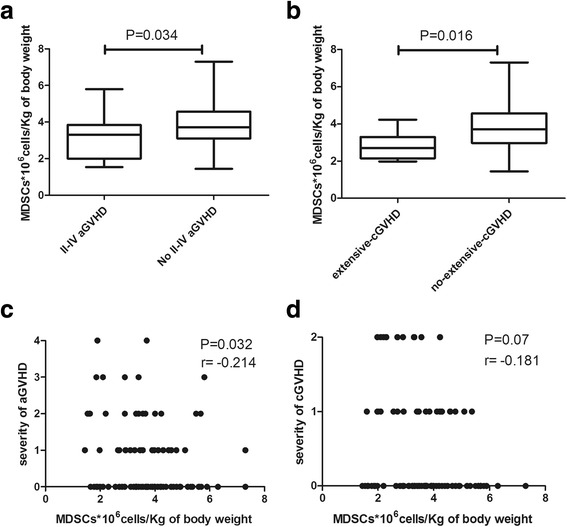
Box plots showing the absolute number of infused MDSCs with respect to II–IV aGVHD (**a**) and extensive cGVHD (**b**). *Bars* represent the median values, whereas *whiskers* represent the minimum and maximum. The severity of aGVHD (**c**) and cGVHD (**d**) were plotted against the number of infused MDSCs. The grade for severity of cGVHD included limited and extensive cGVHD (*1* = limited, *2* = extensive, respectively). *r* and *P* values were calculated using Spearman’s correlation test

The impact of graft contents on the incidence of GVHD was further analyzed. The results showed that patients who developed no II–IV aGVHD received statistically higher numbers of MDSCs than patients who did (median, 3.73 × 10^6^ cells/kg; range, 1.4 to 7.3 compared to 3.31 × 10^6^ cells/kg; range, 1.5 to 5.8, respectively, Fig. 3a, *P* = 0.034). Similarly, patients who developed no extensive cGVHD received significantly higher numbers of MDSCs comparing with patients who developed (median, 3.71 × 10^6^ cells/kg; range, 1.4 to 7.3 compared to 2.7 × 10^6^ cells/kg; range, 1.98 to 4.23, respectively, Fig. 3b, *P* = 0.016). Correlation analysis showed a significantly negative correlation between the numbers of MDSCs infused and the severity of aGVHD (Fig. 3c, *P* = 0.032, *r* = −0.214). And a trend for negative correlation between MDSCs infusion and the severity of cGVHD was also found though without significant difference (Fig. 3d, *P* = 0.07, *r* = −0.181).

While cell doses of other compositions including CD3^+^ T cells and Treg cells did not differ in patients developing acute or chronic GVHD or not (all *P* > 0.05), similarly, no significant correlation was found between the numbers of other cellular components and acute or chronic GVHD (all *P* > 0.05).

### Infections post-transplantation

Within the first 100 days post-transplantation, 55 patients developed 86 episodes of bacterial and/or fungal infections. There were 12 and 10 cases of bacterial infections, 7 and 5 cases of fungal infections, and 10 and 11 cases of bacterial and fungal mixed infections, respectively, in G-BM and G-PBSC groups (all *P* > 0.05). One patient died from bacterial infection in G-BM group, while no patient died from infections in G-PBSC group (*P* = 1.000). Within 6 months post-transplantation, 14 (28.6%) patients in G-BM group and 16 (30.8%) patients in G-PB group had CMV viremia (*P* = 0.808); 3 patients in G-BM group and 5 patients in G-PB group had EBV viremia ( *P* = 1.000).

### Survival and relapse

With a median follow-up of 701 days post-transplantation, 76 patients were alive. Eleven patients in the G-BM and 14 in the G-PBSC group died. Causes of death included leukemia relapse (*n* = 7 vs 8), GVHD (*n* = 1 vs 3), and infections (*n* = 3 vs 3). Ten patients died of TRM, with 4 patients in G-BM group, and 6 patients in G-PBSC group (*P* = 0.333). Nineteen patients experienced relapse, as a result of 9 in G-BM group and 10 in G-PBSC group. Of the 19 relapse patients, 3 patients abandoned treatment and the other 16 patients were treated with chemotherapy, donor lymphocyte infusion, or second allo-HSCT. Seven cases achieved CR after treatment.

**Fig. 4:**
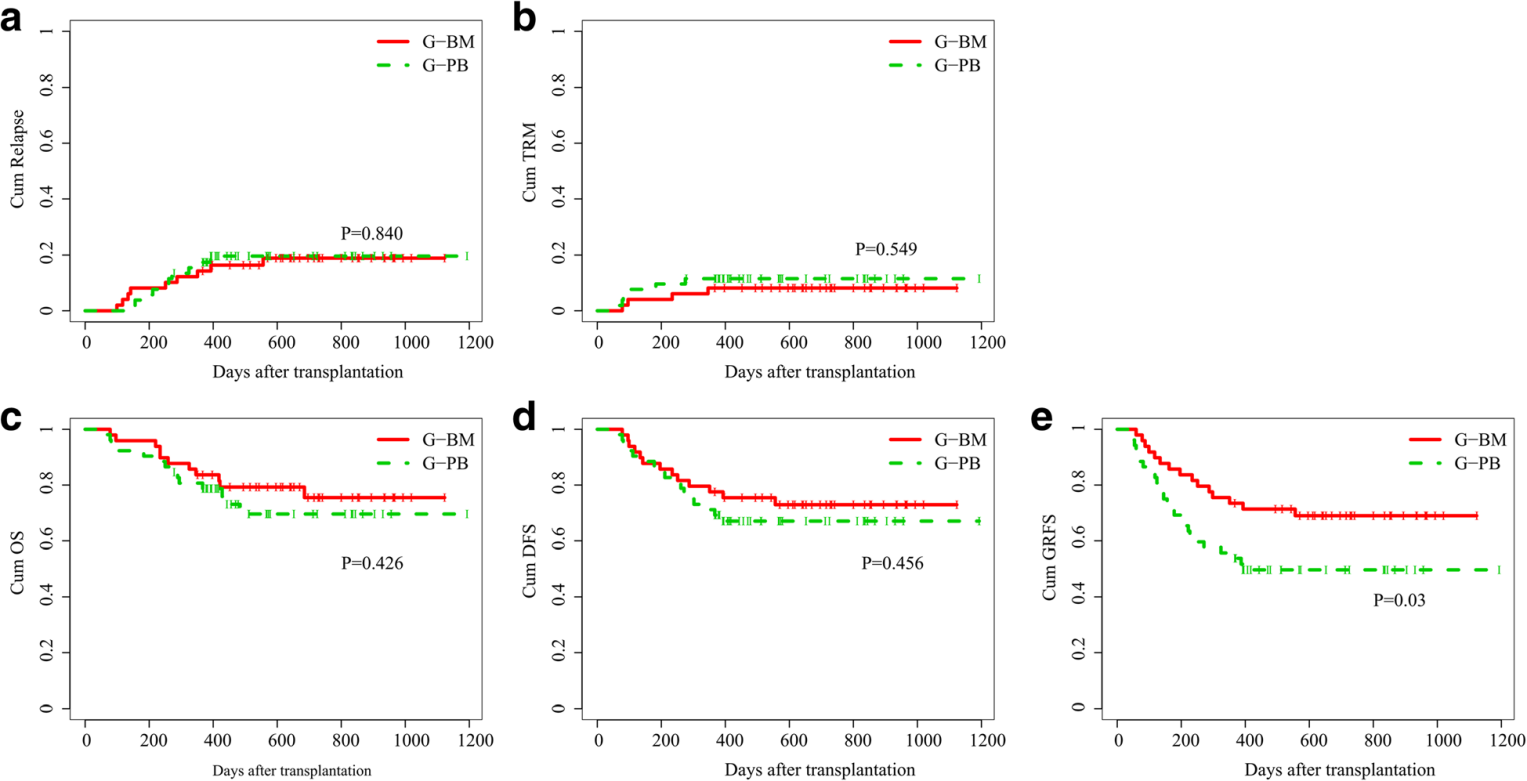
Cumulative incidence of 3-year **a** relapse, **b** TRM, **c** OS, **d** DFS, and **e** GRFS. The 3-year cumulative incidence of relapse were 18.9% ± 5.8% and 19.6% ± 5.7% in G-BM and G-PBSC groups (*P* = 0.840), and TRM were 8.2% ± 4.0% and 11.5% ± 4.5%, respectively, in G-BM and G-PBSC groups (P = 0. 549). The 3-year cumulative OS were 75.5% ± 6.7% and 69.6% ± 7.1%, respectively, in the G-BM and G-PBSC group (*P* = 0.426), and DFS were 72.9% ± 6.4% and 67.1% ± 6.6%, respectively, in the G-BM and G-PBSC groups (*P* = 0.456). The 1-year cumulative GRFS were 73.5% ± 6.3% vs 55.8% ± 6.9%, respectively, in the G-BM and G-PBSC groups (*P* = 0.049), and 2- and 3-year cumulative GRFS were 69.0% ± 6.7% vs 49.7% ± 7.0%, respectively, in the G-BM and G-PBSC groups (*P* = 0.03)

The 3-year cumulative incidence of relapse was 18.9% ± 5.8% and 19.6% ± 5.7%, respectively, in the G-BM and G-PBSC groups (Fig. 4a, *P* = 0.840), and TRM were 8.2% ± 4.0% and 11.5% ± 4.5%, respectively, in the G-BM and G-PBSC groups (Fig. 4b, *P* = 0.549). The 3-year cumulative OS were 75.5% ± 6.7% and 69.6% ± 7.1%, respectively, in the G-BM and G-PBSC groups (Fig. 4c, *P* = 0.426), and DFS were 72.9% ± 6.4% and 67.1% ± 6.6%, respectively, in the G-BM and G-PBSC groups (Fig. 4d, *P* = 0.456). The G-BM group showed significantly higher probability of GRFS than the G-PBSC group (73.5% ± 6.3% vs 55.8% ± 6.9% at 1 year, *P* = 0.049; 69.0% ± 6.7% vs 49.7% ± 7.0% at 2 and 3 years, *P* = 0.03, respectively, Fig. 4e).

Risk factors for survival and relapse are presented in Table 3. In multivariate analysis, high risk of disease (HR = 2.31; 95% cumulative incidence (95% CI), 1.09–4.87; *P* = 0.028) was associated with a lower DFS. MDSC cell dose below the median of 3.65 × 10^6^ cells/kg (HR = 3.49; 95% CI, 1.77–6.9; *P* < 0.001), older recipient age (HR = 2.02; 95% CI, 1.07–3.8; *P* = 0.039), and high risk of disease (HR = 2.14; 95% CI, 1.14–4.02; *P* = 0.018) were significantly associated with a lower GRFS. All factors studied were not significantly associated with relapse or OS in the univariate and multivariate analyses (all *P* > 0.05) (Table 3). Both CD3^+^ T cells and Treg cells did not influence the risk of relapse, OS, DFS, and GRFS. And no significant correlation between MDSC cell dose and relapse was found (*P* > 0.05), which indicated that higher content of MDSCs did not increase the incidence of relapse in this study.

**Table 3:** Univariate and multivariate analyses for relapse, OS, DFS, and GRFS

Risk factors	Relapse		OS		DFS		GRFS	
	Univariate	Multivariate (*P* (HR, 95% CI))	Univariate	Multivariate (P (HR, 95% CI))	Univariate	Multivariate (*P* (HR, 95% CI))	Univariate	Multivariate (*P* (HR, 95% CI))
Female vs male	NS	NS	NS	NS	NS	NS	NS	NS
Patient age, ≥33 vs <33 years	NS	NS	NS	NS	NS	NS	NS	*P* = 0.039 (2.02, 1.07–3.8)
ALL vs AML	NS	NS	NS	NS	NS	NS	NS	NS
High risk vs standard risk	NS	NS	NS	NS	*P* = 0.03	*P* = 0.028 (2.31, 1.09–4.87)	NS	*P* = 0.018 (2.14, 1.14–4.02)
G-PBSC vs G-BM	NS	NS	NS	NS	NS	NS	*P* = 0.03	*P* = 0.071 (1.82, 0.95–3.49)
CD3^+^ T, ≥166 × 10^6^ vs <166 × 10^6^ cells/kg	NS	NS	NS	NS	NS	NS	NS	NS
Treg, <5.2 × 10^6^ vs ≥5.2 × 10^6^ cells/kg	NS	NS	NS	NS	NS	NS	NS	NS
MDSCs, <3.65 × 10^6^ vs ≥3.65 × 10^6^ cells/kg	NS	NS	NS	NS	NS	NS	*P* < 0.001	*P* < 0.001 (3.49,1.77–6.90)

*OS* overall survival, *DFS* disease-free survival, *GRFS* GVHD-free/relapse-free survival, *ALL* acute lymphoblastic leukemia, *AML* acute myelogenous leukemia, *TNC* total nucleated cells

## Discussion

In the past two decades, unstimulated BM grafts have lost its use as the optimal source of HSCs because G-PBSC grafts has faster hematopoietic reconstruction. However, G-PBSC grafts have its shortage of increased GVHD compared with unstimulated BM grafts. Recently, some studies suggested that G-BM grafts had similar hematopoietic reconstruction, and less cGVHD, compared with G-PBSC grafts. Serody et al. [24] and Morton et al. [25] reported that G-BM grafts showed significantly less cGVHD and aGVHD, respectively, and comparable engraftment to G-PBSC grafts. The recent investigation from a larger prospective randomized study showed that both the neutrophil and platelet recovery were statistical 3 days later, and G-BM grafts resulted in a significantly lower cGVHD compared to G-PBSC grafts [26]. In this study, our results showed similar engraftment of neutrophil and platelet and a significantly lower cGVHD and aGVHD in G-BM group compared with that in G-PBSC group. Meanwhile, the presented study was compared with the historical data of patients who underwent unstimulated BM grafts in our institutions. The results showed that G-BM grafts had significantly faster engraftment in both neutrophil and platelet engraftment and a trend of lower acute and chronic GVHD compared with unstimulated BM grafts.

A variety of factors affect the incidence of GVHD, such as graft source, donor source, HLA compatibility, and GVHD prophylaxis [27–31]. Each graft source has its own components and show different immunological properties, which affect the incidence and severity of GVHD [10, 32, 33]. Though G-PBSC grafts contain 4- to 10-fold more T lymphocytes compared with un-BM grafts, the incidence and severity of aGVHD is surprisingly low and even comparable with that in unstimulated BM grafts [1], which is considered as a result of G-CSF-induced immune regulation.

G-CSF induces immune tolerance through a variety of mechanisms including modulation on graft contents [34, 35]. MDSCs are a heterogeneous population with a potent immunosuppressive activity [11]. Recently, several experimental and clinical evidences suggested that MDSCs might play an immunoregulatory role in GVHD. Highfill et al. [13] demonstrated that adoptive transfer of MDSCs could inhibit the severity and mortality of GVHD. Vendramin et al. [14] reported that higher doses of monocytic MDSCs (M-MDSCs) in the G-PBSC grafts were associated with less aGVHD. Guan et al. [36] reported that the number of G-MDSCs in PB of patients at the preconditioning time point was negatively correlated with aGVHD. As the cellular effectors of aGVHD are mainly cytotoxic T lymphocytes and natural killer cells [37, 38], MDSCs are able to inhibit alloreactive responses mediated by T lymphocytes and NK cells through a variety of mechanisms, including l-arginine depletion by arginase 1 and the inducible nitric oxidase (iNOS), generation of reactive oxygen species, release of transforming growth factor-beta (TGF-beta) and IL-10, cysteine sequestration, and regulatory T (Treg) cell induction [11, 39–41]. In this study, our results were in line with the studies from Highfill et al. [13] and Vendramin et al. [14] showing that MDSC infusions were negatively correlated with incidence and severity of GVHD. Moreover, we found that G-BM grafts were associated with a significantly higher frequency of MDSCs, which might associate with its lower GVHD.

It is known that GVL effect mainly depends on T cell activity. So, we reasoned whether the higher MDSC content in the G-BM grafts increase the incidence of leukemia relapse because of its probable downregulated T cell responses. Animal models had demonstrated that the adoptive transfer of MDSCs can result in the successful control of GVHD without compromising GVT effects [13, 42]. In this study, the results revealed no significant difference in the relapse of G-BM and G-PBSC group and no significant correlation between the number of MDSCs infused and leukemia relapse. Similarly, Vendramin et al. [14] reported that the number of M-MDSCs infused did not correlate with tumor relapse. In addition, we also compared infections within the first 100 days post-transplantation, and the result showed no significant difference between the two groups, which might attribute to comparable engraftment with G-PBSC grafts. Meanwhile, it also indicated that higher MDSC content did not increase the infection rate.

GRFS is the primary endpoint of this research. Our results revealed that G-BM grafts showed significantly lower GVHD but similar hematopoietic reconstruction as well as relapse compared with G-PBSC grafts. So we questioned whether these results come to superior GRFS. As a result, the G-BM group showed significantly higher probability of 1-, 2-, and 3-year GRFS than the G-PBSC group. Considering the small sample size of this study, larger clinical trials need to be conducted as well as with further follow-up is wanted. In addition, BM grafts contain stromal cells such as mesenchymal stem cells (MSC), which show immunomodulatory effect on various immune cells. Whether stromal cells in G-BM grafts is associated with its lower GVHD compared with G-PBSC grafts need further research.

## Conclusions

G-BM grafts lead a better GRFS and less GVHD associated with a higher MDSC content compared with G-PBSC grafts. And MDSCs might play the immunoregulatory role in GVHD.

## Abbreviations

95% CI: 95% cumulative incidence
ALL: Acute lymphoid leukemia
allo-HSCT: Allogeneic hematopoietic stem cell transplantation
AML: Acute myeloid leukemia
ANC: Absolute neutrophil count
BM: Bone marrow
Bu: Busulfan
CFSE: 5, 6-carboxy-fluorescein diacetate succinimidyl ester
cGVHD: Chronic graft-versus-host disease
CMV: Cytomegalovirus
CR1: First complete remission
Cy: Cyclophosphamide
DFS: Disease-free survival
EBV: Epstein-Barr virus
G-BM: Mobilized bone marrow
G-CSF: Granulocyte colony-stimulating factor
G-PBSC: Mobilized peripheral blood stem cells
GRFS: GVHD-free/relapse-free survival
GVHD: Graft-versus-host disease
GVL: Graft-versus-leukemia
HLA: Human leukocyte antigen
HR: Hazard ratio
HSCs: Hematopoietic stem cells
HVOD: Hepatic veno-occlusive disease
IFI: Invasive fungal infection
IMC: Immature myeloid cells
iNOS: Inducible nitric oxidase
MDSCs: Myeloid-derived suppressor cells
M-MDSCs: Monocytic myeloid-derived suppressor cells
NK: Natural killer
OS: Overall survival
PB: Peripheral blood
TBI: Total body irradiation
TGF-beta: Transforming growth factor-beta
Th2: T helper type 2
TNC: Total nucleated cells
TRM: Transplant-related mortality
